# The Type III Secretion System plasmid pPHDPT3 of *Photobacterium damselae* subsp. *piscicida* is stable in Australian isolates due to conserved non-repetitive genomic architecture

**DOI:** 10.1093/femsle/fnag074

**Published:** 2026-06-24

**Authors:** Oleksandra Rudenko, Laura Baseggio, Andrew C Barnes

**Affiliations:** School of the Environment, The University of Queensland, Brisbane QLD 4072, Australia; School of the Environment, The University of Queensland, Brisbane QLD 4072, Australia; School of the Environment, The University of Queensland, Brisbane QLD 4072, Australia; School of Agriculture and Food Sustainability, The University of Queensland, Brisbane QLD 4072, Australia

**Keywords:** *Photobacterium damselae* subsp. *piscicida (Pdp)*, pPHDPT3, pPHDP10, plasmid repeats, plasmid dimers, putative resolvase

## Abstract

*Photobacterium damselae* subsp*. piscicida* (*Pdp*) is a host-adapted primary pathogen impacting finfish aquaculture worldwide, whose virulence evolution is driven by the mobilome. The pPHDPT3 plasmid encoding a type III secretion system is critical for *Pdp* virulence but unstable *in vitro* in European and Japanese isolates. Here we show that a stable ancestral variant is conserved in Australian isolates. The elusive pPHDPT3 variant has undergone gene loss and accumulated a ∼5.5 kb quadruple direct repeat, which could explain plasmid loss via the classic dimer catastrophe scenario. In addition, we hypothesize that a 189-aa serine recombinase encoded within this repeat, and also on pPHDP10 plasmid, may act as a plasmid resolvase, with its frequent loss further exacerbating the dimer catastrophe.

## Teaser

A plasmid encoding the type III secretion system in *Photobacterium damselae* subsp. *piscicida* shows substantial sequence divergence and markedly different stability outside the host in Australian and overseas isolates.

## Introduction

*Photobacterium damselae* subsp*. piscicida* (*Pdp*), a member of the family *Vibrionaceae*, is a highly adapted pathogen of marine finfish with global distribution. Evolution from its free-living sister sub-species *Photobacterium damselae* subsp*. damselae* was shaped by mobile genetic elements (Baseggio et al. 2021), with the best-characterized virulence factors being the AIP56 toxin and the siderophore piscibactin, encoded on the pPHDP10 and pPHDP70 plasmids, respectively (do Vale et al. 2005, Osorio et al. 2015).

More recently, Abushattal et al. (Abushattal et al. 2022) demonstrated a strong association between *Pdp* virulence and the presence of another ∼ 130 kb plasmid, pPHDPT3, which encodes a type III secretion system (T3SS). This finding was linked to the discovery of *in vitro* instability of pPHDPT3 in *Pdp* isolates from Europe and Japan. Consistent with selective advantage in the host conferred by delivery of effector proteins into the host cells via T3SS (needle-like structure, injectisome) (Wagner et al. 2018), all primary isolation colonies recovered from fish tissues contained pPHDPT3. Remarkably, a single laboratory subculture of these colonies on non-selective agar was sufficient to cure the plasmid at 50% frequency (Abushattal et al. 2022). The *in vitro* elusiveness of pPHDPT3 in European and Japanese isolates is reflected in its absence or only partial representation in their genome assemblies (Abushattal et al. 2022), with the genome of the PP3 isolate sequenced by Abushattal et al. (Abushattal et al. 2022), comprising, to our knowledge, the only complete sequence of unstable pPHDPT3 currently available. Importantly, the spontaneous pPHDPT3-deficient mutants were tested in an *in vivo* challenge and were almost avirulent. The rapid loss of pPHDPT3 and associated virulence *in vitro* was reported to be a generalized feature among *Pdp* strains (Abushattal et al. 2022).

In Australia, *Pdp* affects yellowtail kingfish, *Seriola lalandi*, and isolates of *Pdp* collected during 2015–16 outbreaks on kingfish farms were extensively investigated both *in vitro* and *in vivo* (Baseggio et al. 2021, 2022, Rudenko et al. 2023). These isolates, QMA0505 and QMA0506, were shown to represent a distinct and highly virulent evolutionary lineage, with pPHDPT3 being one of the five plasmids found in their genomes (Baseggio et al. 2021, 2022). Contrary to what might be expected for a virulence-promoting genetic element requiring host selective pressure for maintenance, pPHDPT3 was retained without any change in the genomes of eleven clones generated during QMA0505 knockout mutagenesis (Rudenko et al. 2023). These laboratory mutants not only underwent numerous subculturing steps involving a single-colony isolation, but they were also subjected to multiple stressful conditions, including high osmotic stress, electroporation, antibiotic selection, sucrose counter-selection, multiple freezing/storage/and recovery from -80°C (Rudenko et al. 2023).

The first goal of the present study was to verify the pPHDPT3 stability in the Australian *Pdp* population over time and empirically confirm the absence of its rapid loss *in vitro*. The second goal was to identify genetic differences leading to pPHDPT3 (in) stability.

## Methods

### Bacterial strains, culture, and colony-PCR

*In vitro* stability of pPHDPT3 in Australian *Pdp* strains was investigated as described in Abushattal et al., using two epidemiologically independent isolates from yellowtail kingfish, *Seriola lalandi*: AS-16–0540-1 (QMA0505) isolated in 2016 (Baseggio et al. 2021) and QMA0776 isolated in 2022. Isolates were recovered from −80°C glycerol stocks by streaking on Tryptic Soy Agar supplemented with 0.5% NaCl (TSA-1) and grown at 25°C or 18°C for 48 h or 72 h, respectively. Fourteen isolated colonies were picked from each plate (*n* = 56), re-suspended in 30 μl of nuclease-free water, and 1.5 μl of these suspensions were used as PCR template. The latter was added to 23.5 μl master mix containing 12.5 μl OneTaq® Quick-Load® 2X Master Mix with Standard Buffer (NEB), 0.5 μl of 10 μM vscJ_PP3_F/R primers (Abushattal et al. 2022), and 10 μl nuclease-free water. Amplification was performed as follows: 10 min of initial denaturation at 94°C, followed by 30 cycles of 30 s of denaturation at 94°C, 30 s of primer annealing at 52°C, and 30 s extension at 68°C. Moreover, 14 isolated colonies from each first sub-culture plate were re-streaked on fresh TSA-1 plates, incubated as above, and two isolated colonies from each second sub-culture plate were PCR-screened for presence of T3SS gene *vscJ* as above (*n* = 112). This experiment was performed twice with a total of 112 and 224 colonies screened for the loss of pPHDPT3 after the first and second sub-culture, respectively.

### Comparative sequence analysis

Complete genome of QMA0505 isolate (Baseggio et al. 2021) and PP3 isolate (Abushattal et al. 2022) were downloaded from NCBI GenBank database (CP061854-60 and SRHT02000010-SRHT02000010 accession numbers, respectively). The genome of QMA0776 was sequenced and assembled as previously described for QMA0505 (Baseggio et al. 2021), and deposited under JBRTRI010000002.1-JBRTRI010000007.1 accession numbers. Geneious Prime 2023.0.4 (https://www.geneious.com) was used to visualize and analyse the data, and to generate Figs. 2–5. Sequence alignments were performed using the Clustal Omega 1.2.3 (Sievers et al. 2011) plugin. Sequence repeats over 800 bp in length were identified using the Repeat Finder 1.0.1 (Biomatters Ltd.) plugin. Homology search was performed via BLAST+ (Camacho et al. 2009) against NCBI nr database via NCBI website or custom databases created in Geneious. Prediction of protein domains was carried out using SMART 9.0 (Letunic et al. 2020). To detect pPHDP10 multimers, Nanopore reads from QMA0776 isolate (SRR35779798) were mapped onto trimeric assembly of the pPHDP10 contig (JBRTRI010000007) using Minimap 2.24 and Geneious aligners. *Dif* sites recognized by XerC/XerD recombinases were predicted by FIMO (Find Individual Motif Occurrences, MEME Suite) (Grant et al. 2011) against the *dif* consensus ‘ATTTA CATAAT NNNNNN TTATGTTAAAT’ (11 bp XerC arm—6 bp spacer—11 bp XerD arm (Carnoy and Roten 2009) under p threshold 1-E6, and exported into Geneious to locate and analyse the candidates.

## Results and discussion

### Australian population of *Pdp* harbours pPHDPT3 which is temporarily conserved and stably maintained *in vitro*

To assess the stability of pPHDPT3 in the Australian *Pdp* population, isolate QMA0776, collected during a 2022 outbreak, was subjected to whole-genome sequencing, and a complete assembly was generated (accession JBRTRI010000002.1-JBRTRI010000007.1). This genome was compared with that of the reference isolate QMA0505 from a 2015 outbreak (Baseggio et al. 2021). The comparison revealed an unchanged genetic architecture comprising two chromosomes of ∼3.2 Mb and 1.1 Mb, and five plasmids: three virulence plasmids pPHDP10, pPHDP70, pPHDPT3, and two uncharacterized plasmids of ∼23 and 37 kb.

This genome architecture is largely conserved in the PP3 isolate, a representative strain carrying the unstable pPHDPT3 (the only complete sequence of this variant available to date), apart from an additional ∼286 kb plasmid encoding tetracycline resistance [4]. Four of the plasmids identified in QMA0505 correspond to single contigs of equivalent size in the PP3 assembly, while the fifth (∼23 kb) unnamed plasmid is represented by three contigs (SRHT02000001.1–SRHT02000003.1). This indicates an overall conserved plasmidome between these isolates, with significant sequence divergence and instability in PP3 cultures appearing to be restricted to pPHDPT3.

The alignment of pPHDPT3 sequences from QMA0505 and QMA0776 did not identify any major changes, with only 39 SNPs detected within the 136.3 kb sequence located in either non-coding regions or in transposable element (TE) genes, with the only exceptions being 6 non-synonymous SNPs in a gene encoding the T3SS regulon anti-activator, ExsD (IC627_21 965). This demonstrates pPHDPT3 conservation in natural *Pdp* population over time in Australia.

**Figure 1:**
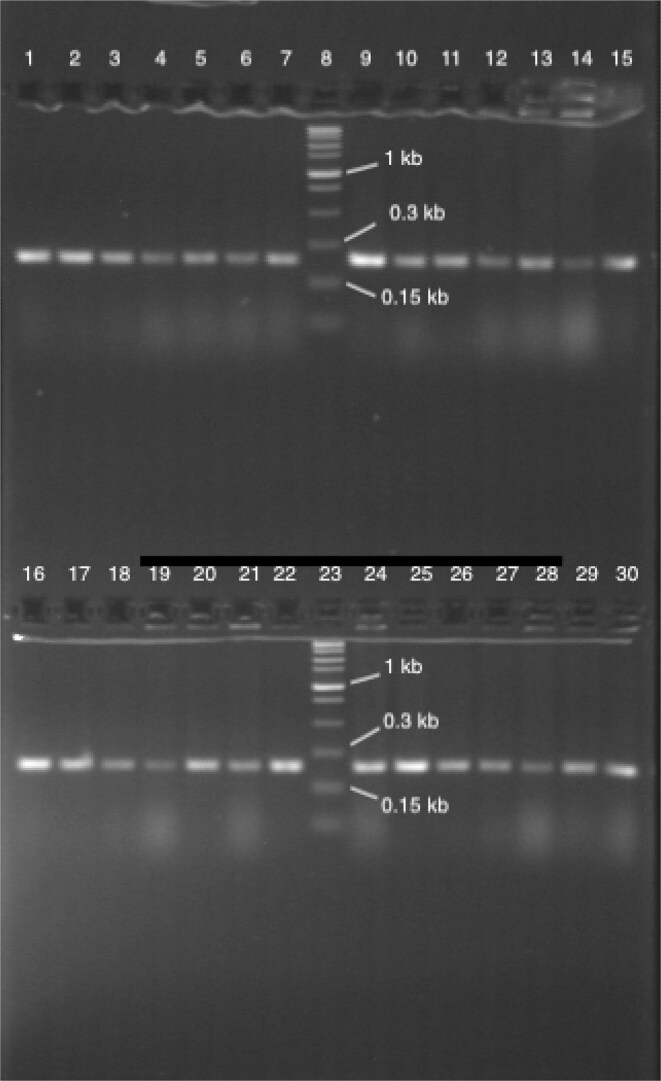
Example gel of the colony PCR-screening for retention of pPHDPT3. Lanes 8 and 23 contain Fast DNA Ladder (NEB) with 10, 5, 3, 2, 1.5, 1 (reference), 0.766, 0.5, 0.3, 0.15, 0.05 kb bands, other lanes contain 239 bp amplicons derived from a single-colony PCR with vscJ_PP3_F/R primers [3].

*In vitro* stability of the Australian pPHDPT3 previously suggested by absence of any mutations in sequenced QMA0505 mutagenesis clones (Rudenko et al. 2023) was further confirmed here by targeted colony PCR screening for retention of pPHDPT3 during sub-culturing, using T3SS gene *vscJ-*specific primers (Abushattal et al. 2022). The total of 112 colonies screened after the first subculture of QMA0505 and QMA0776 (recovered from frozen stocks), and 224 colonies screened after the second subculture, were all *vscJ*-positive indicating no plasmid loss (Fig. 1). This is in complete contrast to reported overseas isolates showing loss of pPHDPT3 at 50% frequency after the first subculture of primary isolation colonies recovered directly from fish tissues (Abushattal et al. 2022).

### The elusive pPHDPT3 has undergone gene loss and acquired large repeats that may account for its instability

Sequence comparison between the stable Australian pPHDPT3 variant, represented by QMA0505, and unstable European/Japanese pPHDPT3 variant, represented by PP3, revealed significant differences, which may explain the marked differences in plasmid stability *in vitro*.

#### Sequence shared between QMA0505 and PP3 (∼87.5 kb)—T3SS and remnants of T4SS

**Figure 2:**
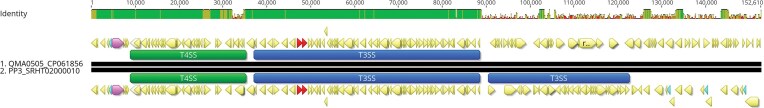
Nucleotide alignment comparing stable and unstable pPHDPT3: sequence from the Australian isolate QMA0505 (CP061856, top) and Japanese isolate PP3 (SRHT02000010, bottom). T4SS and T3SS—type 3 and type 4 secretion system gene clusters respectively. Highlighted annotations are: *parA* and *parB* plasmid partitioning genes (red), 189 aa resolvase (light blue), and transposase (purple) from Tn3 transposon. Identity bar shows nucleotide pairwise identity, where green represents 100% identity, greenish-brown denotes 31–99% identity, and red represents < 30% identity.

About 68% of the pPHDPT3 sequence (∼87.5 kb) is highly similar in both isolates. This shared part is comprised of a type IV secretion system (T4SS) gene cluster, followed by the T3SS cluster, and multiple transposable elements (TE) genes outside and inside these clusters (Fig. 2, left). Most of the T4SS genes represent a truncated conjugal transfer cluster from a plasmid of ancestral sister sub-species *Photobacterium damselae* subsp*. damselae* (*Pdd*) (Abushattal et al. 2022).

However, QMA0505 harbours an additional T4SS segment containing four extra annotations (IC627_22110–25), including a gene encoding a VirB5 protein (IC627_22 110) reported to be critical for virulence pilus formation (Yuan et al. 2005). These sequences are absent in the *Pdd* ancestor, but the VirB5 gene is found with ∼70% identity in *Vibrio parahaemolyticus* plasmids (e.g. CP011408; CP047998; CP068651). Copies of these additional T4SS genes are also found on chromosome 2 (IC627_21385–95) and an unnamed 36 kb plasmid (IC627_22935–45) in QMA0505. This may indicate a selective advantage, potentially reflecting the repurposing of T4SS system components for the delivery of effector proteins (Alvarez-Martinez Cristina and Christie Peter 2009).

#### Sequence lost in PP3 (∼50 kb)—multiple genes with unknown function, several with homology to virulence-associated genes

The remaining 47.7 kb of pPHDPT3 in QMA0505, which was lost in PP3 (Fig. 2; ∼47.7 kb, top right), is abundant in TE and genes with unknown function, but several encoded proteins may potentially contribute to the pathogenicity/virulence, including CesT family type III secretion system chaperone (IC627_21 750, 60) (Rahmatelahi et al. 2021), lysozyme inhibitor (IC627_22 385) (Vanderkelen et al. 2012), Hok/Gef family protein (IC627_22 370) (Lobato-Márquez et al. 2016), and a YadA-like family protein (IC627_22 400) (Kajava et al. 2001).

#### Sequences repeats acquired by PP3 (∼45.6 kb)

**Figure 3:**
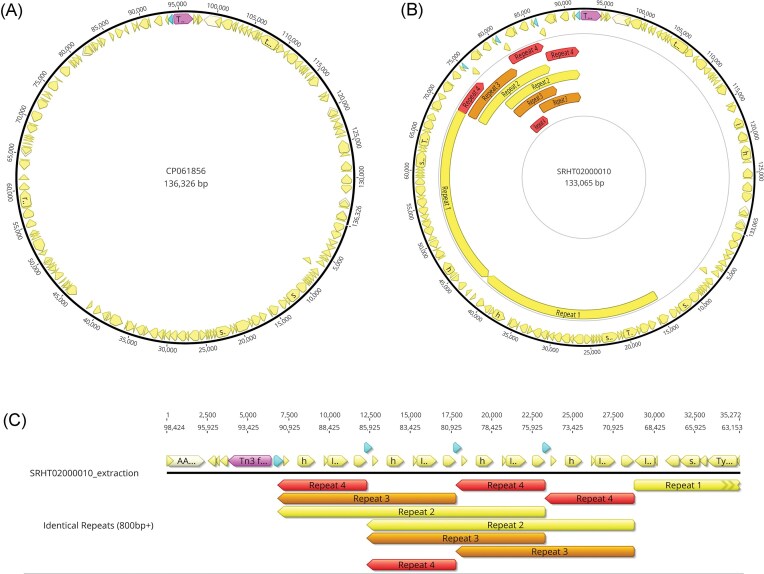
Large sequence repeats on unstable pPHDPT3 from PP3 isolate (B, C), which are absent in stable pPHDPT3 from QMA0505 isolate (A). Repeat 1–oppositely-oriented partial repeat of T3SS cluster; Repeats 2–4–a directly-oriented repeats of the 5.5 kb sequence adjacent to Tn3 family transposase (purple), which encodes a 189 aa resolvase from Tn3 transposon (light blue).

In contrast, the part of pPHDPT3 in PP3 which is absent in QMA0505 (Fig. 2; ∼45.6 kb, bottom right), is likely to have a detrimental effect on both virulence and plasmid stability:

1. Oppositely oriented partial repeat of T3SS cluster with embedded *parAB* genes (∼27.7 kb)
  First, there is a 27.7 kb partial inverted duplication of the T3SS cluster (Fig. 2; Fig. 3, Repeat 1), which appears to be unique to PP3 and closely related strains (Abushattal et al. 2022). Large oppositely oriented repeat regions are prone to frequent inversions via homologous recombination and are therefore generally selected against and rare in bacterial genomes (Achaz et al. 2003). However, although inversions within the repeated T3SS cluster may compromise the T3SS gene expression, they are unlikely to have an immediate effect on plasmid stability. In this context, the partitioning genes, *parA* (E4T26_024 340) and *parB* (E4T26_023 620) are located within the first part of the original T3SS cluster (Fig. 2), which is not duplicated, and consequently cannot be disrupted by the inversions. *ParA/parB* are also flanked by TE which may potentially affect their stability, but this feature is also true for the stable pPHDPT3 version.
2. A directly oriented quadruple repeat of 5.5 kb sequence (∼22 kb)
  The remaining part of the unstable pPHDPT3 consists of a series of directly-oriented repeats encompassing 22 kb: a 5.5 kb sequence is repeated four times, which also creates three identical direct repeats of 11 kb, and two identical direct repeats of 16.5 kb (Fig. 3). As little as 400–800 bp of sequence identity in the direct orientation is generally sufficient for efficient homologous recombination, as reflected by the typical lengths of homology arms used for allelic-exchange mutagenesis, with only rare difficult targets requiring 1–1.2 kb (Faulds-Pain and Wren 2013, Rudenko and Barnes 2018). Consequently, quadruple direct repeat of 5.5 kb will markedly increase the likelihood of homologous recombination for pPHDPT3 (Oliveira et al. 2008).

Homologous recombination between direct repeats on separate plasmid molecules (intermolecular) frequently leads to formation of plasmid dimers which, if not resolved by site-specific recombinases, segregate inefficiently during cell division and can give rise to plasmid-free daughter cells (Bedbrook and Ausubel 1976, Summers et al. 1993). Low-copy number, large virulence plasmids are particularly prone to loss via this phenomenon, known as ‘dimer catastrophe’, and therefore typically encode in *cis* acting effective partitioning systems and multimer resolution enzymes (Nordström and Austin 1989, Sengupta and Austin 2011, Summers 1991). This applies to pPHDPT3, which is ∼130 kb in size, harbours *parA/parB* partitioning genes, and exhibits low-copy number, as indicated by sequencing coverage equivalent to that of the two *Pdp* chromosomes.

When direct repeat copies are misaligned during intermolecular recombination (unequal recombination), a stable plasmid dimer is not generated; rather, the asymmetric cointegrate resolves into two molecules, one losing and the other gaining an additional copy of the repeat (Lovett et al. 1994). Consequently, propagation of the 5.5 kb segment, and its presence in four copies in the elusive pPHDPT3 variant, are consistent with an elevated rate of intermolecular recombination. In the absence of other distinguishing features between stable and unstable pPHDPT3 sequence that could plausibly explain plasmid loss at ∼50% frequency after a single subculture, enhanced multimerization driven by the quadruple 5.5 kb repeat provides a parsimonious explanation for elusiveness of pPHDPT3 in European and Japanese isolates.

### Serine recombinase encoded on pPHDPT3 and pPHDP10: a putative plasmid resolvase

While plasmid dimers form through intermolecular recombination, intramolecular recombination between direct repeats within a single pPHDPT3 molecule would frequently result in deletion of the intervening sequence (Bedbrook and Ausubel 1976, Summers et al. 1993). If this region encodes protein/s critical for plasmid maintenance, its loss would lead to plasmid instability. For this mechanism to apply to pPHDPT3, such protein/s must be encoded as a single-copy locus in the stable variant and within the repeat region in unstable pPHDPT3. Of the 5.5 kb repeated four times in PP3, a 4.5 kb segment is also present in QMA0505 with 98.5% nucleotide identity as a single copy, which contains only five annotated genes, of which just two encode non-TE proteins. The latter are a 399 aa hypothetical protein (IC627_22 280) lacking identifiable functional domains, and a 189-aa recombinase family protein (IC627_22 270) is almost fully composed of a serine recombinase family domain ser_ce (Marshall Stark 2015, Rice 2015).

**Figure 4:**
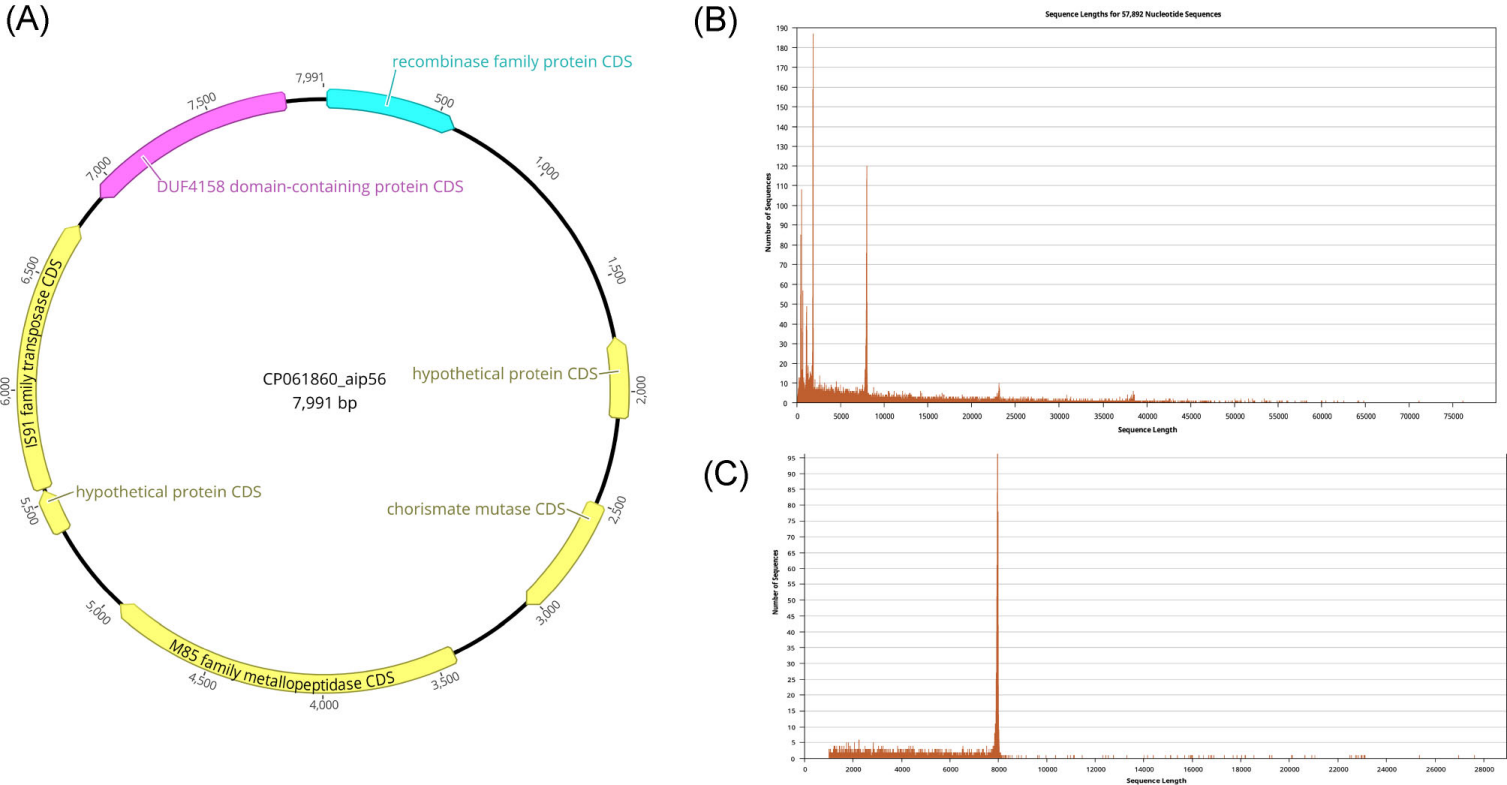
(A) a map of pPHDP10 (monomer) from QMA0505 isolate, highlighted annotations–189 aa resolvase (light blue) and truncated transposase (purple) from Tn3 transposon. (B, C) read lengths graph produced by mapping of long sequencing reads onto a trimeric pPHDP10 contig from QMA0776 genome assembly using Minimap 2 and Geneious alignment algorithms, respectively.

The ancestral members of the serine recombinase family are Tn3-like transposon resolvases that convert cointegrates formed during replicative transposition into separate molecules. However, serine resolvases of transposon origin are often found on plasmids, where they function in plasmid dimer resolution (Marshall Stark 2015, Rice 2015). Indeed, the gene encoding the 189-aa recombinase of *Pdp* was acquired as a part of a Tn3 transposon, as it is found immediately downstream from the Tn3 family transposase gene (IC627_22 265) in pPHDPT3 (Figs. 2 and 3). The identical copy of 189-aa serine recombinase, along with a severely truncated Tn3 transposase annotated as DUF4158 domain protein (IC627_23 240), is also found on pPHDP10, a small virulence plasmid of *Pdp* encoding AIP56 toxin (Fig. 4A). These genes are absent in the ancestral *Pdd* subspecies (Baseggio et al. 2022), but found with 99.6% pairwise nucleotide identity on a 150 kb plasmid (AP025512) from a type strain LMG 20012 of *Vibrio tasmaniensis*.

While pPHDP10 is a small (∼8 kb), moderate copy-number plasmid (6x over chromosome coverage), making its loss via dimer catastrophe unlikely (Summers 1991), it still has an apparent need for an effective multimer resolution system. It belongs to the marine RNA-based (MRB) family of *Vibrionaceaceae* plasmids related to ColE1-type plasmids from *Escherichia coli*, whose replication is primed by RNA (Le Roux et al. 2011, Pan et al. 2010). A strikingly high rate of multimerization was reported historically for ColE1-type plasmids, which is consistent with recent analysis showing unusually high proportion of their long sequencing reads originating from multimers (Kusano et al. 1989, Vaisbourd et al. 2025). While the pPHDPT10 monomer size is ∼8 kb, a pPHDPT10 contig in QMA0776 assembly was ∼24 kb, and sequence examination has revealed a trimeric assembly. When plasmid multimer contigs arise as assembly artifacts (common in long-read-first approaches) (Johnson et al. 2023), read mapping typically reveals only reads ≤ monomer length (Das et al. 2024). In contrast, for the pPHDPT10 trimeric contig, Minimap 2 mapping showed a peak at ∼7.8 kb (monomer), and the additional peaks at ∼23 kb and ∼39 kb, consistent with trimer and pentamer lengths (Fig. 4B). Geneious aligner (more stringent algorithm), mapped 9670 reads of the monomer size, but 70 multimeric reads were nonetheless mapped, 42 of these also larger than a dimer length (Fig. 4C). In *E. coli*, ColE1-type plasmid multimers are resolved by the chromosomally encoded tyrosine recombinases XerC/XerD [2]. Although a homologous system recognizing the site-specific recombination sites *dif1* and *dif2* on chromosomes 1 and 2, respectively, is present in *Vibrio cholerae* (Val et al. 2008), to date, plasmid dimer resolution by XerC/XerD has not been reported in *Vibrionaceae*. Consistent with this, we identified *xerD* (IC627_12 755), *xerC* (IC627_14 940), and putative *dif* sites with canonical 11 bp XerC/6 bp spacer/11 bp XerD architecture at the chromosome termini (*dif1* ‘TTTTAGCCATA-AAGTGT-ATTGTGCTTT’; *dif2* ‘TATTGTACCTA-TCTTTT-ATTTCCTTAT’), in the QMA0505 genome, but no *dif*-like sites were detected on pPHDP10 or pPHDPT3. This suggests reliance on alternative multimer resolution systems, most likely encoded in *cis*.

**Figure 5:**
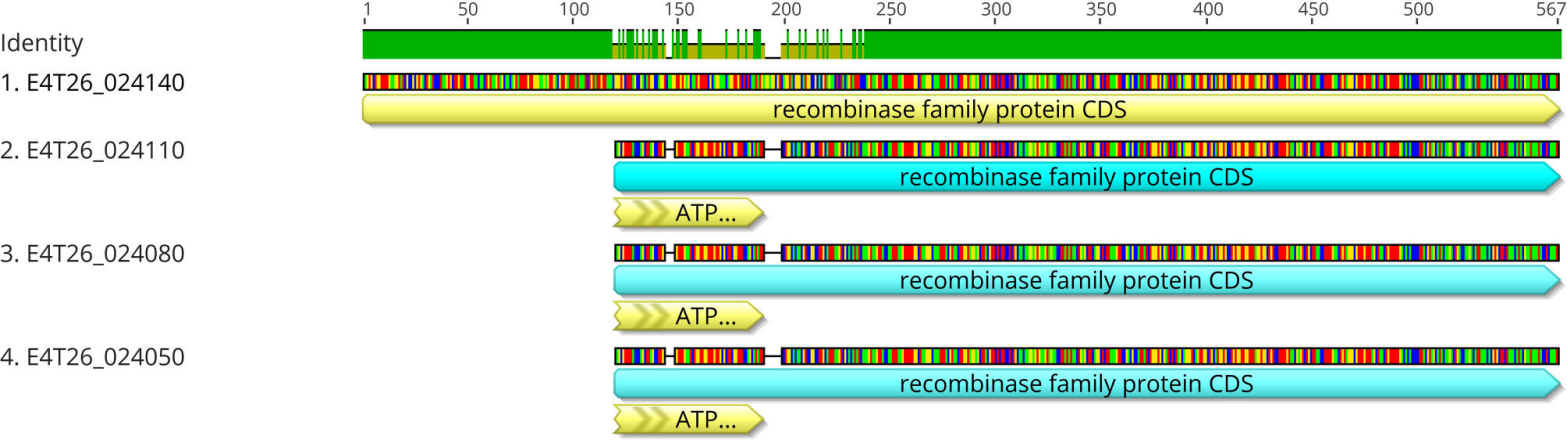
Amino acid alignment of the original 189 aa resolvase from Tn3 transposon in pPHDPT3 of the PP3 isolate (E4T26_024 140) and its three mutated copies found in sequence repeats (E4T26_024 110, E4T26_024 080, E4T26_024 050). Identity bar shows amino acid mean pairwise identity, where green represents 100% identity and greenish-brown denotes 31%–99% identity.

Although experimental functional validation is required, we suggest that 189-aa serine recombinase shared between pPHDP10 and pPHDPT3 functions a *cis*-acting resolvase. In pPHDP10, this is supported by high multimerization, the absence of chromosomal resolvase recognition sites, and a complete lack of alternative *cis*-encoded candidates (only seven genes are encoded, mostly genes with known functions or TE (Fig. 4A). For pPHDPT3, support comes from targeted analysis to identify proteins potentially associated with plasmid maintenance that could be deleted via intramolecular recombination between direct repeats of elusive pPHDPT3. If this hypothesis is correct, quadruple direct repeat of 5.5 kb may not only promote multimerization, but also decrease the ability to separate the multimers due to the loss of a resolvase. In this respect, truncation/deletion of the original recombinase copy (E4T26_024 140) would be sufficient, as three repeated copies are significantly mutated and likely yield a deficient protein (Fig. 5).

## Conclusion

The T3SS-encoding low-copy-number plasmid pPHDPT3 differs between Australian and overseas *Pdp* isolates: while Australian isolates retain a stable ancestral variant, a derived elusive variant that was described in European and Japanese isolates, has undergone gene loss and accumulated several large sequence repeats. The only identifiable sequence divergence plausibly underlying the high frequency of rapid *in vitro* loss is a ∼5.5 kb quadruple direct repeat, likely promoting plasmid dimer catastrophe via the classic mechanism and, additionally, through loss of a putative resolvase. Resulting T3SS-free daughter cells may have a fitness advantage outside the host, such as reduction of energetic cost; alternatively, the repeat may simply be a product of non-adaptive evolution.

## Data Availability

The complete genome assembly of *Photobacterium damselae* subsp*. piscicida* isolate QMA0776 was deposited under NCBI WGS project JBRTRI01 (BioProject PRJNA662633, BioSample SAMN52394294, Nanopore and Illumina reads at SRA—SRR35779798 and SRR35779799, respectively).

## References

[bib1] Abushattal S, Vences A, Osorio CR. A highly unstable and elusive plasmid that encodes the type III secretion system is necessary for full virulence in the marine fish pathogen *Photobacterium damselae* subsp. *piscicida*. Int J Mol Sci. 2022;23:4729. 10.3390/ijms23094729 35563122 PMC9105992

[bib2] Achaz G, Coissac E, Netter P et al. Associations between inverted repeats and the structural evolution of bacterial genomes. Genetics. 2003;164:1279–89. 10.1093/genetics/164.4.1279 12930739 PMC1462642

[bib3] Alvarez-Martinez Cristina E, Christie Peter J. Biological diversity of prokaryotic type IV secretion systems. Microbiol Mol Biol Rev. 2009;73:775–808. 10.1128/MMBR.00023-09 19946141 PMC2786583

[bib4] Baseggio L, Rudenko O, Buller N et al. Complete, closed and curated genome sequences of *Photobacterium damselae* subsp. *piscicida* isolates from Australia indicate mobilome-driven localized evolution and novel pathogenicity determinants. Microb Genom. 2021;7:000562. 10.1099/mgen.0.000562 33885359 PMC8208687

[bib5] Baseggio L, Rudenko O, Engelstädter J et al. The evolution of a specialized, highly virulent fish pathogen through gene loss and acquisition of host-apecific survival mechanisms. Appl Environ Microb. 2022;88:e0022222. 10.1128/aem.00222-22 PMC9317898 35862683

[bib6] Bedbrook JR, Ausubel FM. Recombination between bacterial plasmids leading to the formation of plasmid multimers. Cell. 1976;9:707–16. 10.1016/0092-8674(76)90134-3 797459

[bib7] Camacho C, Coulouris G, Avagyan V et al. BLAST+: architecture and applications. BMC Bioinf. 2009;10:1–9. 10.1186/1471-2105-10-421 PMC2803857 20003500

[bib8] Carnoy C, Roten CA. The dif/Xer recombination systems in proteobacteria. PLoS One. 2009;4:e6531. 10.1371/journal.pone.0006531 19727445 PMC2731167

[bib9] Das S, Delamare-Deboutteville J, Barnes AC et al. Extraction of high-molecular-weight DNA from Streptococcus spp. for nanopore sequencing in resource-limited settings. Microbiologyopen. 2024;13:e1432. 10.1002/mbo3.1432 39166362 PMC11336654

[bib10] do Vale A, Silva MT, dos Santos NM et al. AIP56, a novel plasmid-encoded virulence factor of *Photobacterium damselae* subsp. *piscicida* with apoptogenic activity against sea bass macrophages and neutrophils. Mol Microbiol. 2005;58:1025–38. 16262788 10.1111/j.1365-2958.2005.04893.x

[bib11] Faulds-Pain A, Wren BW. Improved bacterial mutagenesis by high-frequency allele exchange, demonstrated in Clostridium difficile and Streptococcus suis. Appl Environ Microb. 2013;79:4768–71. 10.1128/AEM.01195-13 PMC3719504 23728809

[bib12] Grant CE, Bailey TL, Noble WS. FIMO: scanning for occurrences of a given motif. Bioinformatics. 2011;27:1017–8. 10.1093/bioinformatics/btr064 21330290 PMC3065696

[bib13] Johnson J, Soehnlen M, Blankenship HM. Long read genome assemblers struggle with small plasmids. Microb Genom. 2023;9:001024. 10.1099/mgen.0.001024 PMC10272865 37224062

[bib14] Kajava AV, Cheng N, Cleaver R et al. Beta-helix model for the filamentous haemagglutinin adhesin of Bordetella pertussis and related bacterial secretory proteins. Mol Microbiol. 2001;42:279–92. 10.1046/j.1365-2958.2001.02598.x 11703654

[bib15] Kusano K, Nakayama K, Nakayama H. Plasmid-mediated lethality and plasmid multimer formation in an Escherichia coli recBC sbcBC mutant. Involvement of RecF recombination pathway genes. J Mol Biol. 1989;209:623–34. 10.1016/0022-2836(89)90000-4 2685325

[bib16] Le Roux F, Davis BM, Waldor MK. Conserved small RNAs govern replication and incompatibility of a diverse new plasmid family from marine bacteria. Nucleic Acids Res. 2011;39:1004–13. 20923782 10.1093/nar/gkq852 PMC3035462

[bib17] Letunic I, Khedkar S, Bork P. SMART: recent updates, new developments and status in 2020. Nucleic Acids Res. 2020;49:D458–60. 10.1093/nar/gkaa937 PMC7778883 33104802

[bib18] Lobato-Márquez D, Díaz-Orejas R, García-del Portillo F. Toxin-antitoxins and bacterial virulence. FEMS Microbiol Rev. 2016;40:592–609. 10.1093/femsre/fuw022 27476076

[bib19] Lovett ST, Gluckman TJ, Simon PJ et al. Recombination between repeats in Escherichia coli by a recA-independent, proximity-sensitive mechanism. Molec Gen Genet. 1994;245:294–300. 10.1007/BF00290109 7816039

[bib20] Marshall Stark W. The Serine Recombinases. Mobile DNA III. 2015;73–89. 10.1128/9781555819217.ch3

[bib21] Nordström K, Austin SJ. Mechanisms that contribute to the stable segregation of plasmids. Annu Rev Genet. 1989;23:37–69. 10.1146/annurev.ge.23.120189.000345 2694936

[bib22] Oliveira PH, Lemos F, Monteiro GA et al. Recombination frequency in plasmid DNA containing direct repeats—predictive correlation with repeat and intervening sequence length. Plasmid. 2008;60:159–65. 10.1016/j.plasmid.2008.06.004 18647618

[bib23] Osorio CR, Rivas AJ, Balado M et al. A transmissible plasmid-borne pathogenicity island confers piscibactin biosynthesis in the fish pathogen *Photobacterium damselae* subsp. *piscicida*. Appl Environ Microb. 2015;81:5867–79. 10.1128/AEM.01580-15 PMC4551267 26092457

[bib24] Pan L, Leung PC, Gu JD. A new ColE1-like plasmid group revealed by comparative analysis of the replication proficient fragments of Vibrionaceae plasmids. J Microbiol Biotechnol. 2010;20:1163–78. 10.4014/jmb.1003.03007 20798577

[bib25] Rahmatelahi H, El-Matbouli M, Menanteau-Ledouble S. Delivering the pain: an overview of the type III secretion system with special consideration for aquatic pathogens. Vet Res. 2021;52:146. 10.1186/s13567-021-01015-8 34924019 PMC8684695

[bib26] Rice PA. Serine Resolvases. Microbiol Spectr. 2015;3:Mdna3–0045-2014. 10.1128/microbiolspec.MDNA3-0045-2014 PMC5659196 26104713

[bib27] Rudenko O, Barnes AC. Gibson Assembly facilitates bacterial allelic exchange mutagenesis. J Microbiol Methods. 2018;144:157–63. 10.1016/j.mimet.2017.11.023 29196271

[bib28] Rudenko O, Baseggio L, McGuigan F et al. Transforming the untransformable with knockout minicircles: high-efficiency transformation and vector-free allelic exchange knockout in the fish pathogen *Photobacterium damselae*. Microbiologyopen. 2023;12:e1374. 10.1002/mbo3.1374 37642481 PMC10441182

[bib29] Sengupta M, Austin S. Prevalence and significance of plasmid maintenance functions in the virulence plasmids of pathogenic bacteria. Infect Immun. 2011;79:2502–9. 10.1128/IAI.00127-11 21555398 PMC3191983

[bib30] Sievers F, Wilm A, Dineen D et al. Fast, scalable generation of high-quality protein multiple sequence alignments using Clustal Omega. Mol Syst Biol. 2011;7:539. 10.1038/msb.2011.75 21988835 PMC3261699

[bib31] Summers DK, Beton CWH, Withers HL. Multicopy plasmid instability: the dimer catastrophe hypothesis. Mol Microbiol. 1993;8:1031–8. 10.1111/j.1365-2958.1993.tb01648.x 8361350

[bib32] Summers DK. The kinetics of plasmid loss. Trends Biotechnol. 1991;9:273–8. 10.1016/0167-7799(91)90089-Z 1367567

[bib33] Vaisbourd E, Bren A, Alon U et al. Preventing multimer formation in commonly used synthetic biology plasmids. ACS Synth Biol. 2025;14:1309–15. 10.1021/acssynbio.4c00508 40101192 PMC12012879

[bib34] Val ME, Kennedy SP, El Karoui M et al. FtsK-dependent dimer resolution on multiple chromosomes in the pathogen Vibrio cholerae. PLoS Genet. 2008;4:e1000201. 10.1371/journal.pgen.1000201 18818731 PMC2533119

[bib35] Vanderkelen L, Ons E, Van Herreweghe JM et al. Role of lysozyme inhibitors in the virulence of avian pathogenic Escherichia coli. PLoS One. 2012;7:e45954. 10.1371/journal.pone.0045954 23049900 PMC3458809

[bib36] Wagner S, Grin I, Malmsheimer S et al. Bacterial type III secretion systems: a complex device for the delivery of bacterial effector proteins into eukaryotic host cells. FEMS Microbiol Lett. 2018;365:fny201. 10.1093/femsle/fny201 30107569 PMC6140923

[bib37] Yuan Q, Carle A, Gao C et al. Identification of the VirB4-VirB8-VirB5-VirB2 pilus assembly sequence of type IV secretion systems. J Biol Chem. 2005;280:26349–59. 10.1074/jbc.M502347200 15901731

